# Study on Process Optimization and Mechanism of Quartz Sand Acid Leaching and Purification

**DOI:** 10.3390/ma19173604

**Published:** 2026-08-25

**Authors:** Ziyang Bai, Huan Xiong, Yupeng He, Youjun Lu, Bo Ma, Wenzhou Sun, Haibei Shi, Jianjun Wang, Maohui Li

**Affiliations:** 1 School of Materials Science and Engineering, North Minzu University, Yinchuan 750021, China; 20247330@stu.nmu.edu.cn (Z.B.);; 2 Ningxia SiFusion Co., Ltd., Yinchuan 750021, China; 3 Helanshan Laboratory, Yinchuan 750021, China

**Keywords:** impurity occurrence, acid washing process, gradient experiment, impurity removal mechanism

## Abstract

The occurrence characteristics of impurities and the precise technologies used for their removal are the main factors limiting the high-end applications of quartz sand. To simultaneously achieve ultrahigh purity and a high yield while incorporating a sustainable waste acid treatment strategy, quartz sand (SP1) from India was selected as the research material. A stepwise experimental scheme involving single-acid and mixed-acid treatments was adopted to optimize the purification conditions, incorporating a complete process of pretreatment, acid leaching, and post-treatment along with a defined waste liquid management method. The raw SP1 quartz samples and samples treated under the optimal acid-washing conditions were characterized through qualitative and quantitative analyses, including X-ray diffraction, Raman spectroscopy, scanning electron microscopy, and inductively coupled plasma mass spectrometry. The optimal result for the single-acid system was obtained by treating quartz sand with 70 mL of hydrofluoric acid at 120 °C for 2 h, resulting in a purity of 99.9963%. For the mixed-acid system, the volume ratio of hydrofluoric acid, hydrochloric acid, and nitric acid was maintained at 1:6:2, and acid leaching was performed at a constant temperature of 120 °C for 5 h. Under these conditions, the aluminum and iron contents were reduced to 15.23 and 0.15 μg/g, respectively, while the purity and production yield of the acid-washed quartz sand reached 99.9964% and 82.11%, respectively. Based on comprehensive consideration of the purification effectiveness and process economy, an HF:HCl:HNO_3_ volume ratio of 1:6:2 and an acid-washing reaction time of 5 h were selected, providing a stable purity exceeding 99.996%. This study provides a theoretical and technical basis for the standardized and sustainable production of high-purity quartz sand.

## 1. Introduction

Quartz crucibles are essential consumables for producing monocrystalline silicon ingots through the Czochralski method [1]. As the primary raw material used to fabricate quartz crucibles, the quality of high-purity quartz sand directly determines the purity, corrosion resistance, and overall performance of the crucibles. The rapid growth of the global semiconductor industry has created unprecedented demand for high-purity quartz (HPQ). However, the worldwide supply of HPQ is constrained by the geological scarcity of premium quartz deposits and persistent technical difficulties in removing trace lattice impurities and fluid inclusions. The global market for semiconductor-grade HPQ sand has grown continuously, driven by the expansion of wafer fabrication and photovoltaic manufacturing. China’s demand for HPQ sand in semiconductor applications has increased rapidly, with annual global HPQ consumption reaching approximately 3.6 million tons by 2024, of which China accounts for a considerable proportion (~1.5 million tons). However, domestic production capacity remains insufficient to satisfy high-end demand, and quartz mineral raw materials produced domestically have relatively low purity, contrasting sharply with the stringent purity requirements of advanced semiconductor applications [2,3]. Consequently, the market remains heavily dependent on overseas supplies [4,5]. This gap between supply and demand primarily arises from two major challenges in purifying ordinary quartz sand to semiconductor-grade standards. First, the quality of raw quartz ore mined in China is often inadequate, and quartz deposits explicitly suitable for producing high-purity quartz sand have not yet been identified in China. Second, existing purification technologies in China cannot effectively process raw quartz ore to achieve the required high-purity grade [6]. The natural coexistence of quartz sand and various mineral impurities in Chinese deposits further complicates purification. These associated minerals frequently have physical and chemical properties very similar to those of quartz. Moreover, fluid/melt inclusions and lattice-defect impurities within these deposits considerably increase the technical complexity of effective separation [7]. Therefore, to broaden the range of raw material sources and develop advanced purification strategies that overcome dependence on a single geographical origin, Indian quartz sand was deliberately selected as the representative test material in this study. The mineralogical characteristics of this material provided a rigorous platform for evaluating the robustness and general applicability of the purification methods. By achieving high-purity upgrading of this challenging exogenous raw material, this study aimed to reduce dependence on a single supplier, diversify the global supply of high-purity quartz raw materials, and establish a technical benchmark for direct conversion.

Acid leaching is widely recognized as a key step in quartz sand purification, although the specific methods vary considerably according to the source material and thus offer substantial opportunities for further research. For example, Larsen et al. used a diluted hydrofluoric acid (HF) solution to selectively remove feldspar minerals from quartz, producing a quartz sand concentrate containing 95.4% silicon dioxide (SiO_2_) [8]. However, the absence of multi-acid synergy prevented deeper purification. Xuesong et al. purified quartz sand using synergistic mixed-acid leaching with hydrochloric, oxalic, and citric acids [9]. Optimization of the process parameters yielded high-purity SiO_2_ with a grade of 99.97%, an iron (Fe) removal efficiency of 73.67%, and a total impurity removal rate of 62.89%. However, environmental concerns related to waste liquid treatment were not addressed, and industrial-scale application faces challenges associated with high costs and precise temperature control. Mixed-acid leaching inherently generates wastewater containing fluorides, heavy metals, and nitrates, presenting a persistent treatment challenge that is also relevant to the present study. Lele et al. determined the optimal acid-leaching conditions using a mixture of HF (0.5 mol/L), hydrochloric acid (HCl, 3.0 mol/L), and nitric acid (HNO_3_, 0.5 mol/L), successfully producing quartz sand with a purity of 99.996% [10]. Nevertheless, the yield of the resulting high-purity quartz sand was not explicitly reported. Overall, previous studies have focused mainly on maximizing purity, whereas important aspects such as production yield, environmental management of spent acids, and the mechanism underlying mixed-acid synergy remain inadequately investigated. These gaps limit the translation of laboratory purification protocols into robust industrial processes.

To address these limitations, the present study offers three original contributions: the co-optimization of purity and yield through systematic single- and mixed-acid leaching experiments, the incorporation of a waste acid treatment scheme to address the environmental bottleneck, and clarification of the impurity removal mechanism, particularly the interaction of HF with lattice-bound Al and Fe species. This work specifically optimized acid formulations and process parameters to achieve both high purity and high mass recovery, characterized the morphological and structural evolution of quartz sand after leaching, and clarified the synergistic functions of HF, HCl, and HNO_3_ in removing trace impurities [11,12]. The primary objectives were to investigate the effects of different acid-leaching protocols on quartz sand purity and the underlying purification mechanisms, thereby contributing to advances in the preparation of high-purity quartz sand and supporting China’s goal of achieving self-sufficiency in this critical material [13,14].

## 2. Materials and Methods

### 2.1. Materials

Raw quartz sand (designated SP1) obtained from a specific location in India was selected as the experimental material. The raw material had a particle size range of 0.1–0.3 mm, was present in powder form, and exhibited a milky-white color. The reagents used were analytical-grade HF (40% *w*/*w*), HCl (36–38% *w*/*w*), and HNO_3_ (65% *w*/*w*).

The occurrence of impurities in SP1 quartz sand was examined using X-ray diffraction (XRD, SmartLab SE, Rigaku Corporation, Tokyo, Japan), Raman spectroscopy (LabRAM HR Evolution, Horiba, Kyoto, Japan), scanning electron microscopy (SEM, ZEISS Sigma 500, Carl Zeiss AG, Oberkochen, Germany), and inductively coupled plasma mass spectrometry (ICP-MS, NexION 1000G, PerkinElmer, Waltham, MA, USA).

The X-ray diffractometer used in this study was equipped with a Cu-target Kα radiation source (λ = 1.5406 Å) and operated at a voltage of 40 kV and a current of 40 mA. The scanning range was 5–80° (2θ), with a scanning speed of 5°/min and a step size of 0.02°. Because the quartz sand samples were nonconductive, the dried samples were uniformly dispersed on conductive carbon tape before SEM analysis, coated with gold, and then placed in the sample chamber for observation. The accelerating voltage was set at 5–20 kV, and the magnification was adjusted according to the analytical requirements. A Raman laser excitation wavelength of 532 nm was used in this study. The laser power was adjusted according to the sensitivity of the samples to prevent thermal damage. The scanning range was 100–3500 cm^−1^, and the spectral resolution was better than 1 cm^−1^. During testing, each sample was placed on a glass slide and flattened. After positioning and focusing using the confocal microscope system, the target microregion was selected for spectral acquisition. Each acquisition lasted 10–30 s, and scanning was repeated 2–3 times to improve the signal-to-noise ratio.

**Figure 1:**
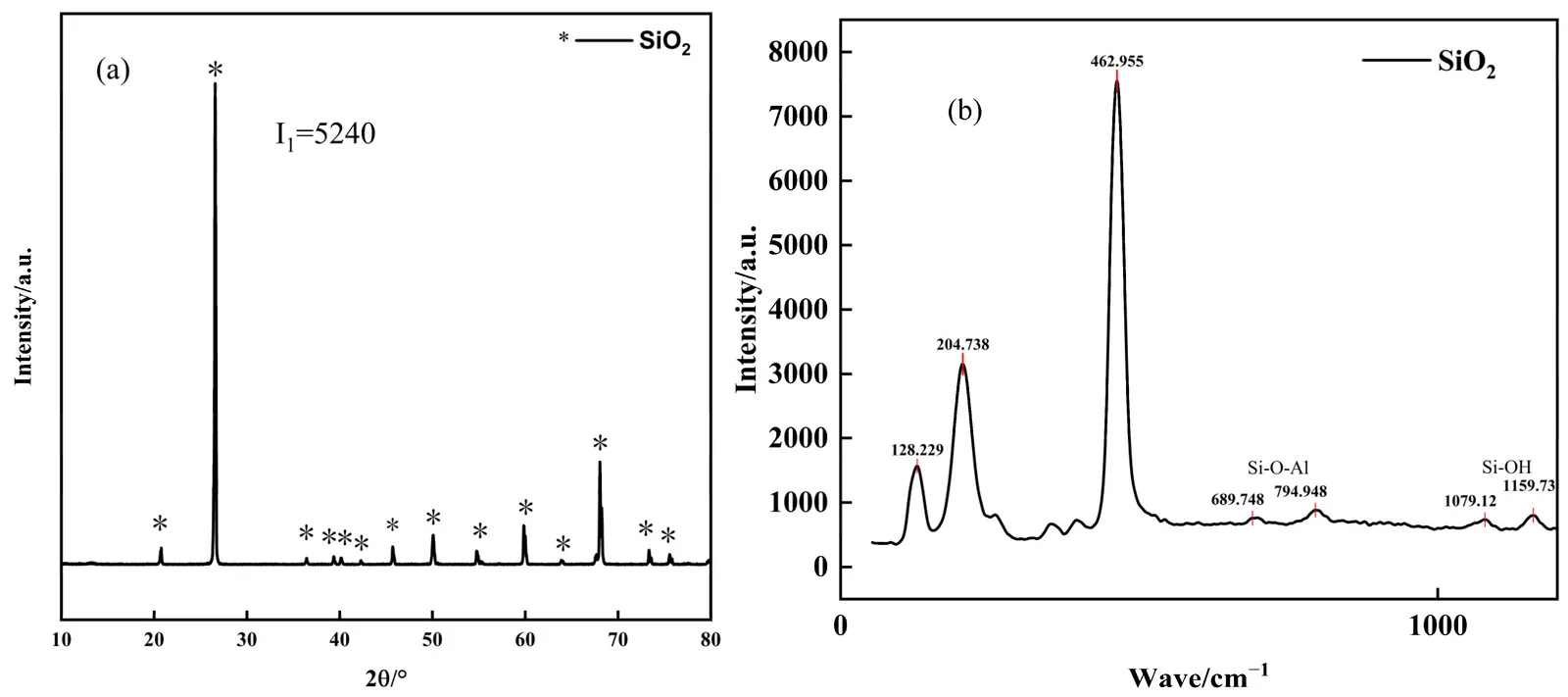
XRD and Raman spectra of SP1 quartz raw sand. (**a**) XRD; (**b**) Raman.

**Figure 2:**
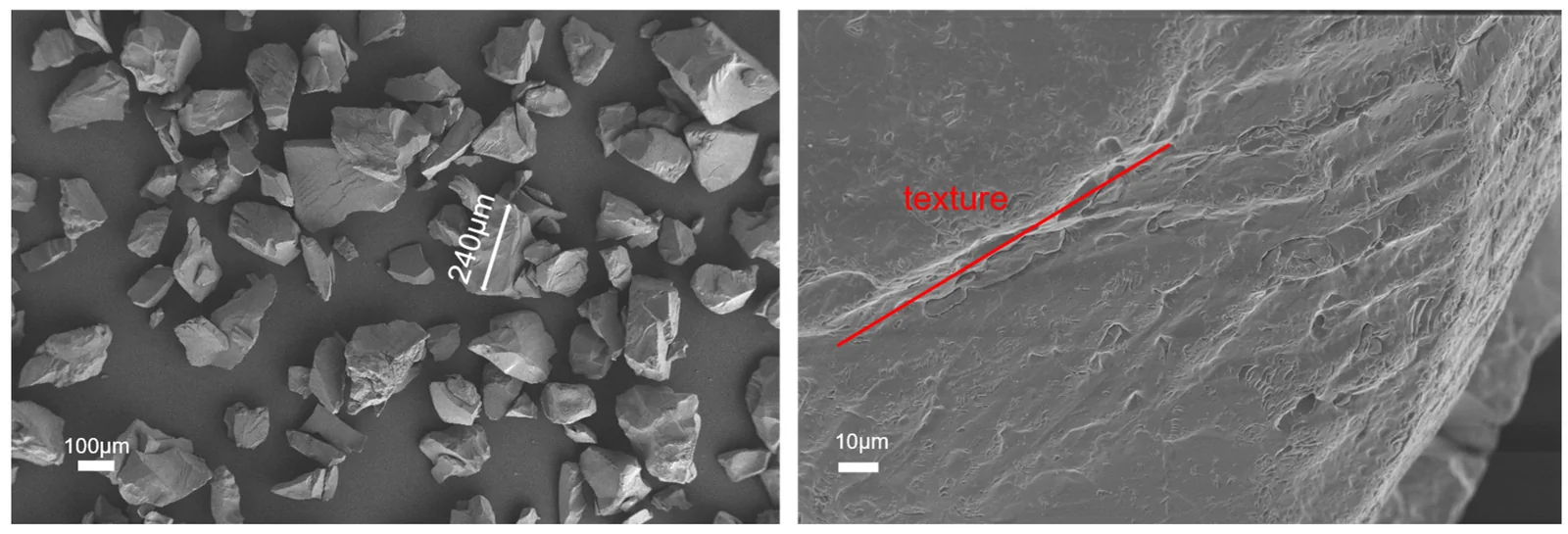
Microscopic morphology of raw SP1 quartz sand.

To verify the structural stability and crystallographic characteristics, profile refinement was performed for the raw quartz sand. As shown in Figure 1a, the diffraction pattern was indexed to a highly ordered hexagonal α-quartz phase (space group P3_2_21). The refined lattice parameters were calculated as a = b = 4.91806 Å and c = 5.40854 Å, with a unit-cell volume of 113.29 Å^3^. The sharp dominant peak at 26.6° exhibited a full width at half maximum (FWHM) of 0.071°, indicating high crystallinity, while whole-pattern refinement produced a satisfactory reliability factor with a residual error (R = 16.25%). These crystallographic parameters confirmed an ordered quartz structure with complete crystallographic characteristics, providing a reliable baseline for comparison with the leached samples. As shown in Figure 1b, the characteristic vibrational modes of α-quartz occurred at 128.299, 204.738, and 462.955 cm^−1^, together with Si–O–Al vibrational peaks at 689.748 and 794.948 cm^−1^. The Si–OH vibrational peaks at 1079.12 and 1159.73 cm^−1^ indicated the incorporation of impurity elements into the lattice through solid solution. As demonstrated in Figure 2, the particle size of the raw quartz sand was well controlled, with no obvious pore defects and a distinct crystalline texture. As shown in Table 1, the principal impurity elements in the raw SP1 quartz sand were Al, Fe, Ti, and alkali metals, and the overall purity was 99.9593%, indicating a relatively high total impurity content.

### 2.2. Acid-Leaching Tests

**Figure 3:**
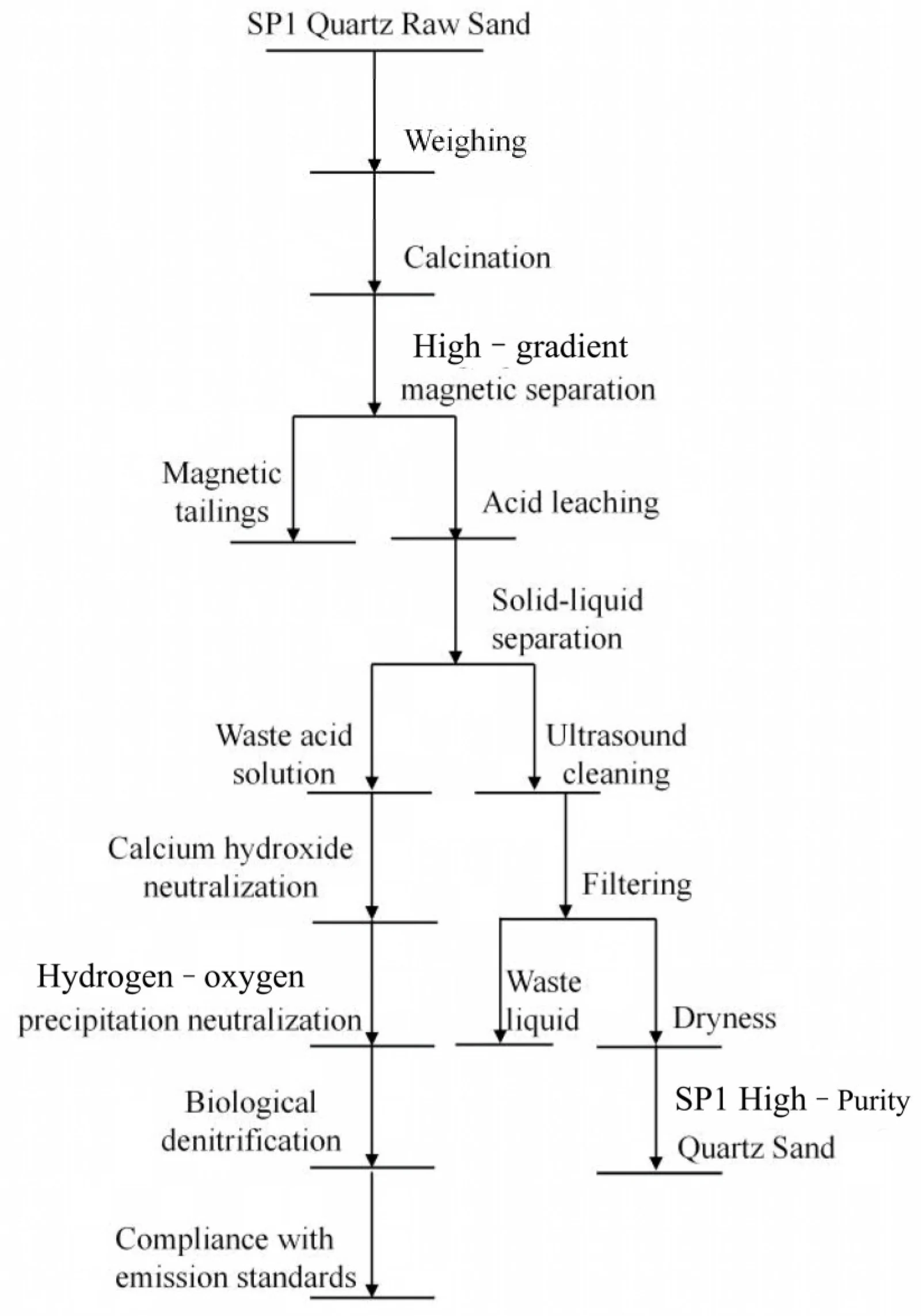
SP1 Sand Production Flow Chart.

The purification process for raw SP1 sand comprised three stages: pretreatment, acid-leaching purification, and post-treatment. The production process for SP1 quartz sand is illustrated in Figure 3. During pretreatment, 20.0000 ± 0.0002 g of SP1 quartz sand was accurately weighed into a quartz crucible and calcined at 1000 °C in a muffle furnace for 120 min. Calcination at 1000 °C was conducted to induce microcracking through the α–β quartz phase transition (~573 °C) and its associated volume change, thereby exposing internal lattice impurities and fluid inclusions to subsequent acid attack. Subsequently, magnetic separation was conducted using a high-gradient magnetic separator at a flow rate of 3 cm/s and a magnetic induction intensity of 1.5 T to remove magnetic impurity minerals. As specified in the detailed acid-leaching parameters in Table 2, stepwise single-acid and mixed-acid leaching systems were used to treat the SP1 quartz sand. The liquid-to-solid ratio for the mixed-acid treatment was 3:1, meaning that the total volume of acid solution was 60 mL. Post-treatment included ultrasonic cleaning at 40 kHz for 30 min, repeated filtration until a neutral pH was reached, and drying at a constant temperature of 105 °C. Furthermore, lime milk was added to the waste acid solution. First, the mixture was neutralized to pH ≈ 5 under thorough stirring to precipitate and immobilize low-solubility calcium fluoride. The system was then further adjusted to weakly alkaline conditions (pH ≈ 8–9) to form hydroxide precipitates, followed by solid–liquid separation. The resulting solid waste was safely disposed of in a hazardous waste landfill. The lime neutralization process effectively immobilized fluoride and precipitated metal hydroxides; however, it did not remove dissolved nitrate ions, which remained in the treated effluent and posed a potential eutrophication risk. The nitrate-rich effluent would require additional, costly biological denitrification steps (e.g., anoxic bioreactors) before discharge to prevent eutrophication. This integrated treatment sequence, while conceptually viable, demands significant energy and infrastructure investment, which is a critical factor for large-scale operations. Ten elements, including Fe, Al, and Ti, were quantitatively analyzed using ICP-MS, with three independent replicate experiments performed to ensure data reliability [15].

### 2.3. Analysis of Quartz Sand Purity

The purity of quartz sand was calculated using Equation (1) [16]. The molecular weights of the impurity-element oxides corresponded to those of 10 major metallic impurities, namely CaO, Na_2_O, K_2_O, Fe_2_O_3_, Al_2_O_3_, Li_2_O, MnO_2_, CuO, TiO_2_, and Cr_2_O_3_.

(1) $${\mathrm{SiO}}_{2}\;\mathrm{purity}=\left(1-\sum \mathrm{Content}\;\mathrm{of}\;\mathrm{impurity}\;\mathrm{elements}\;\times \;\frac{\mathrm{The}\;\mathrm{molecular}\;\mathrm{weight}\;\mathrm{of}\;\mathrm{the}\;\mathrm{oxide}\;\mathrm{corresponding}\;\mathrm{to}\;\mathrm{the}\;\mathrm{impurity}\;\mathrm{element}}{\mathrm{Atomic}\;\mathrm{weight}\;\mathrm{of}\;\mathrm{impurity}\;\mathrm{elements}}\;\right)\times \;100\%$$

### 2.4. Methodological Considerations

Compared with conventional acid-leaching protocols that focus exclusively on the leaching stage, the proposed methodology integrates calcination-induced microcracking, magnetic separation, and a mixed-acid formulation designed to improve impurity removal and mass recovery simultaneously. It also explicitly includes a waste acid neutralization scheme for managing fluoride and heavy-metal contaminants. The current limitations are the high energy demand of the calcination pretreatment, residual nitrate in the effluent that requires further biological denitrification, and validation of the optimized parameters using only one quartz source. Future research should therefore investigate energy-efficient pretreatment alternatives, closed-loop acid regeneration, and validation using multiple quartz sources to evaluate the broader applicability of this approach.

## 3. Results and Discussion

### 3.1. Purity Analysis of Quartz Sand in the Single-Acid System

The impurity contents of quartz sand after single-acid leaching, as measured by ICP-MS, are shown in Table 3.

**Figure 4:**
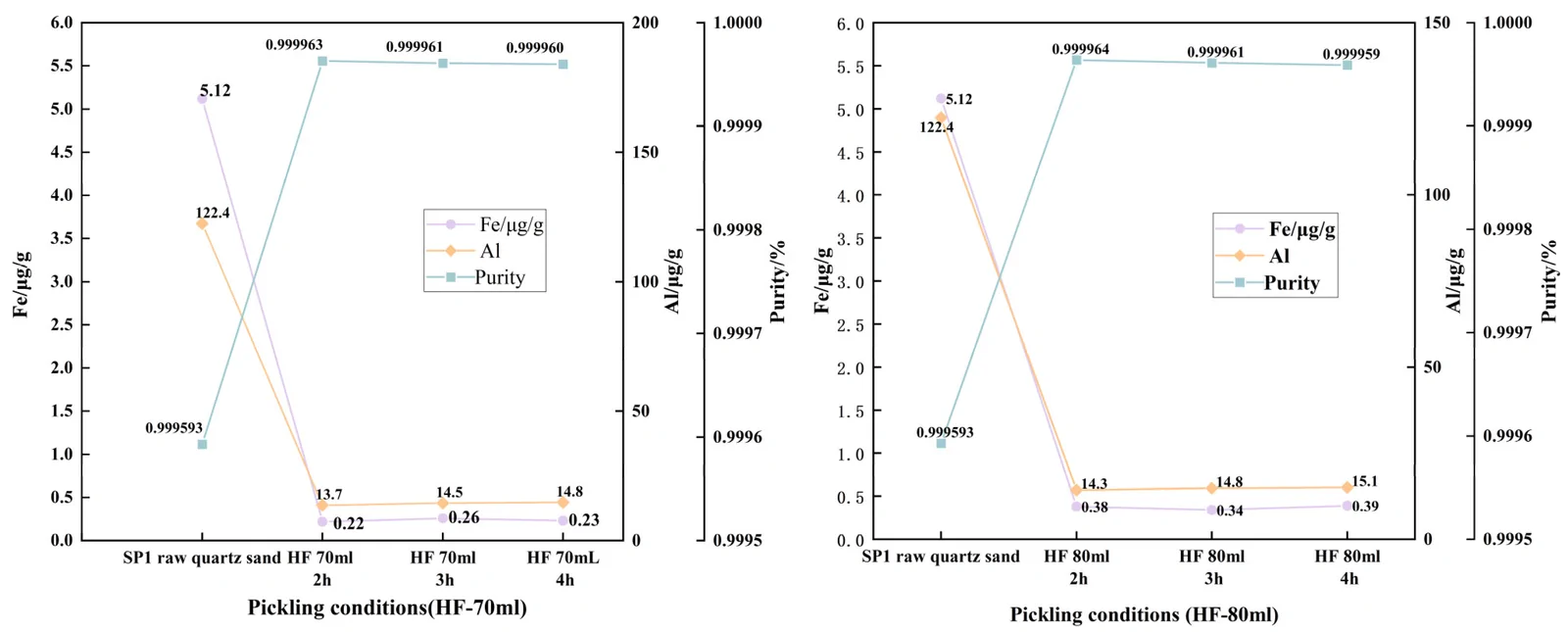
Results obtained under the single-acid leaching conditions.

The results of the acid-leaching experiments are presented in Figure 4 and Table 3. Within the single-acid system, increasing the HF volume from 70 to 80 mL increased the overall purity of quartz sand from 99.9963% to 99.9964%. However, at a constant HF concentration, prolonging the acid treatment gradually reduced the purity. Concurrently, the Fe impurity content, as measured by ICP-MS, increased from 0.22 to 0.38 μg/g. This observation is counterintuitive, as one would expect higher acid dosage to reduce impurity levels. A plausible explanation, based on the known etching behavior of HF, is that highly concentrated HF accelerates the decomposition of silicate inclusions that encapsulate Fe-bearing phases, thereby releasing additional Fe into the solution; however, the excessive dissolution of the quartz matrix may also expose more surface silanol (Si–OH) groups, which could chemically readsorb dissolved Fe ions. Both mechanisms have been proposed in the literature, but the net effect observed here suggests that the release mechanism dominates under our experimental conditions [17]. Furthermore, ICP-MS analysis directly confirms that the Al content increased from 13.7 to 14.3 μg/g as the HF volume was raised from 70 to 80 mL. It hypothesizes that this increase is due to the reaction between HF and Al_2_O_3_ which may generate an AlF_3_-rich passivation layer on the particle surface. Based on the well-documented low solubility and passivating characteristics of AlF_3_ in fluoride-containing acidic media, such a layer would impede the further dissolution of Al-bearing phases, thereby accounting for the higher residual Al content observed in the experiments [18].

(2) $$\mathrm{Productive}\;\mathrm{rate}=\frac{\mathrm{Mass}\;\mathrm{of}\;\mathrm{quartz}\;\mathrm{sand}\;\mathrm{after}\;\mathrm{acid}\;\mathrm{leaching}}{\mathrm{Mass}\;\mathrm{of}\;\mathrm{raw}\;\mathrm{quartz}\;\mathrm{sand}}\times 100\%$$

**Figure 5:**
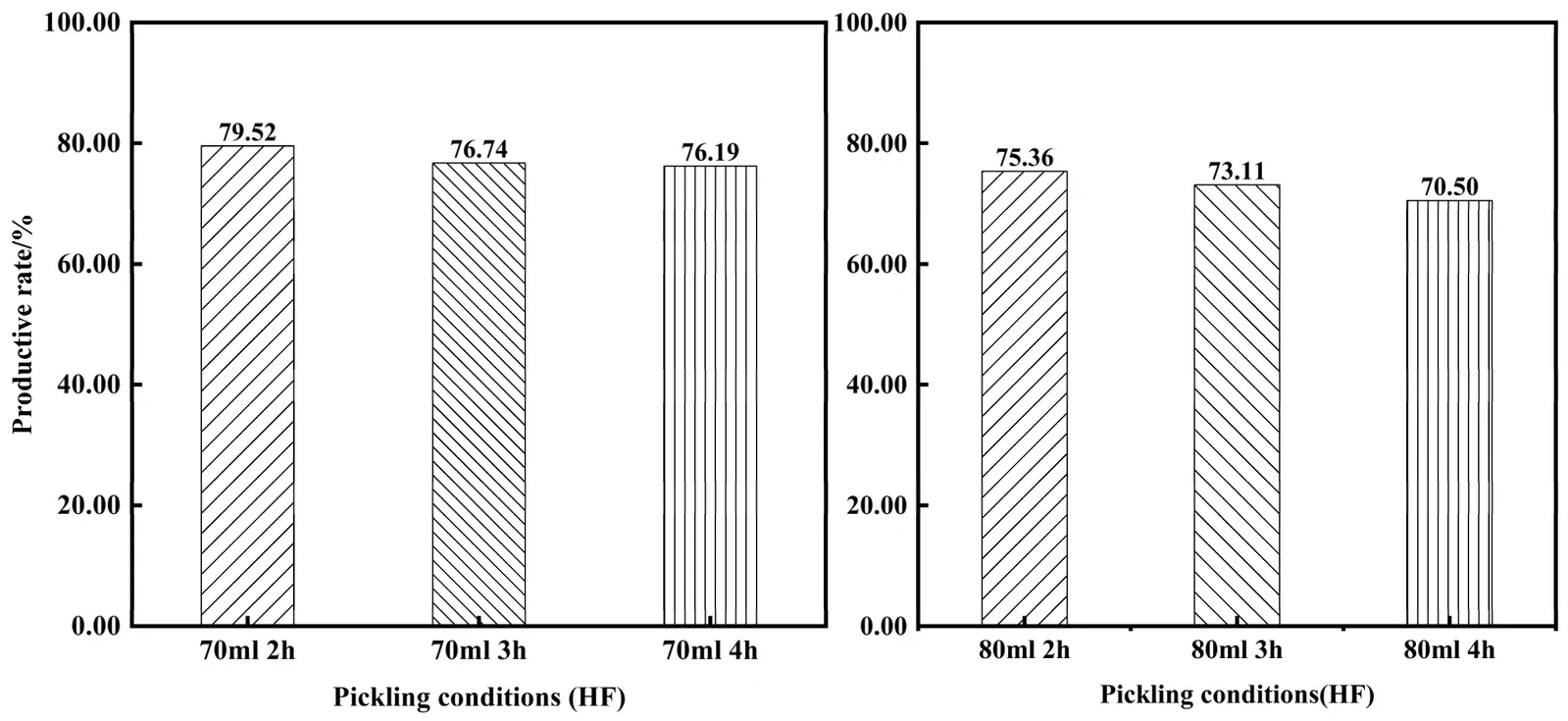
Yield of quartz sand and the contents of selected impurities after single-acid washing.

The quartz sand yield was calculated using Equation (2), and the results are shown in Figure 5. Increasing the treatment time reduced the yield from 79.52% to 76.19%. Similarly, increasing the HF volume from 70 to 80 mL decreased the yield from 79.52% to 75.36%, primarily because of the strong ability of HF to dissolve quartz sand. The primary reaction (Equation (3)) and side reaction (Equation (4)) between HF and quartz sand were irreversible under ambient temperature and pressure conditions. Furthermore, silicon tetrafluoride (SiF_4_), the primary reaction product, existed in a gaseous state at room temperature and readily volatilized [19]. Excess HF resulted in over-etching of the quartz sand and consequently reduced the yield. In addition, HF etching enlarged the microporous structures on the quartz sand surface. This process created mass-transfer channels that enabled subsequent penetration of acids into the quartz crystal lattice, thereby enhancing metathesis reactions or chemical interactions with impurity elements inside the particles [20]. The system optimization experiments demonstrated that maintaining the HF volume at 70 mL and the treatment duration at 2 h produced the optimal balance between purification efficiency and yield in the single-acid system. Therefore, a volume of 70 mL and treatment time of 2 h provided the optimal purification efficacy for quartz sand under single-acid leaching with HF.

(3) $${\mathrm{SiO}}_{2}+4\mathrm{HF}={\mathrm{SiF}}_{4}↑+{2H}_{2}O\;\;\;\;\Delta G^{\theta }=-94.6\;\mathrm{kJ}/\mathrm{mol}$$

(4) $${\mathrm{SiO}}_{2}+6\mathrm{HF}=H_{2}\mathrm{Si}F_{6}+{2H}_{2}O\;\;\;\;\Delta G^{\theta }=+208.5\;\mathrm{kJ}/\mathrm{mol}$$

### 3.2. Purity Analysis of Quartz Sand in the Mixed-Acid System

**Figure 6:**
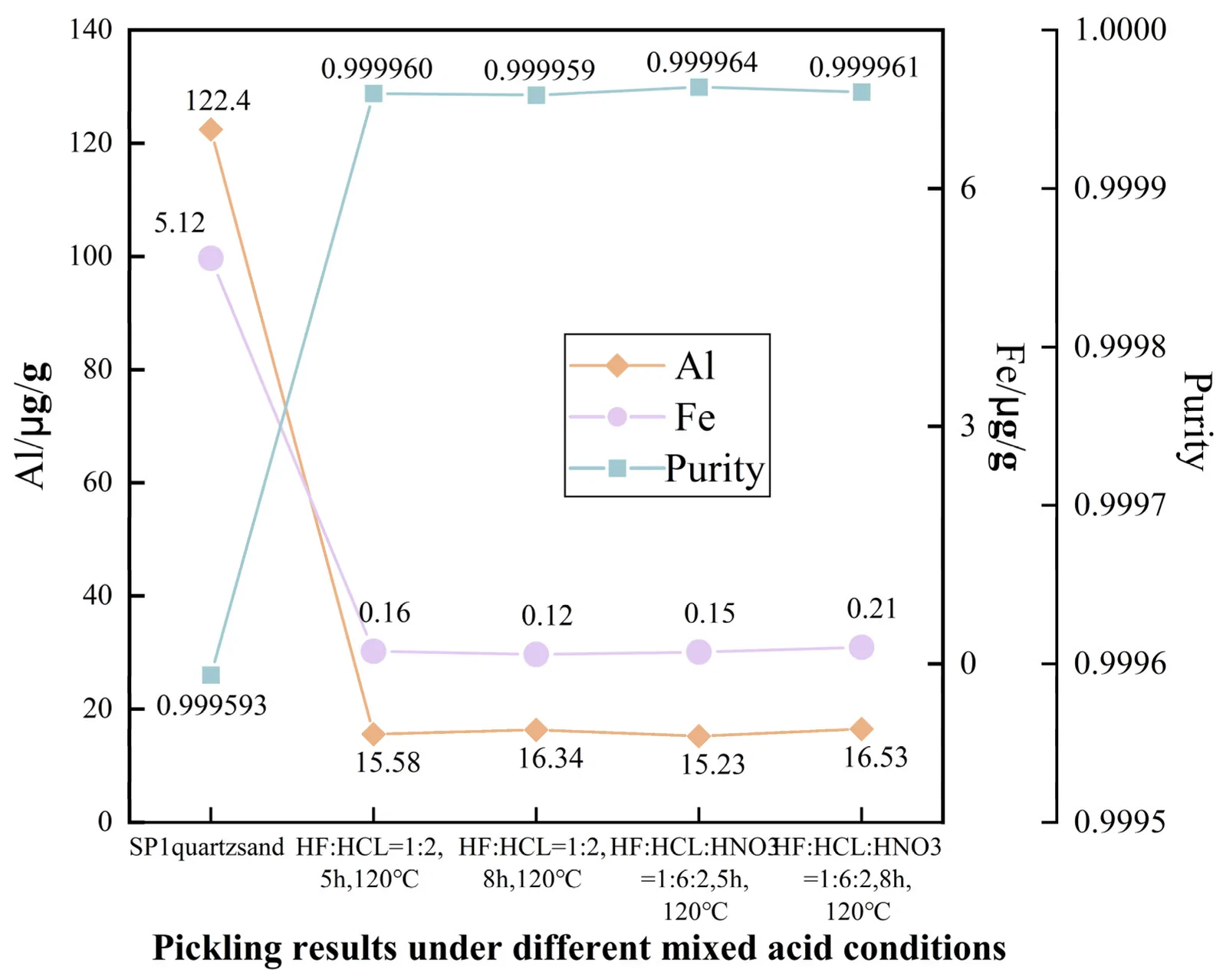
Acid-leaching results under different mixed-acid conditions.

The acid-washing results for raw SP1 quartz sand under different mixed-acid conditions are presented in Figure 6. In the binary mixed-acid system, prolonging the acid-washing duration reduced the Fe content from 0.16 to 0.12 μg/g but increased the Al content from 15.58 to 16.34 μg/g. Consequently, the overall purity declined from 99.996% to 99.9959%. The observed decrease in Fe content is consistent with the known ability of HCl to complex Fe^3+^, promoting the dissolution of iron oxides (Equation (5)). However, the concurrent increase in Al content suggests that Al-bearing phases are less readily attacked under these conditions. A plausible inference, supported by the well-known refractory nature of alumina in acidic media, is that an Al-rich passivation layer may form and hinder further Al dissolution. The apparent discrepancy in dissolution rates between Fe and Al could also be influenced by competitive complexation, wherein the accumulation of dissolved Fe species might impede the dissolution of Al phases; however, this remains a hypothesis that requires further experimental verification. In contrast, Al^3+^ could form insoluble fluoroaluminates in the mixed-acid environment, such as AlF_3_ or hydrated AlCl_3_·nH_2_O, as shown in Equation (6), which would inhibit further reaction. Simultaneously, the accumulation of Fe^2+^/Fe^3+^ could impede Al dissolution through competitive complexation.

(5) $${\mathrm{Fe}}_{2}O_{3}+6\mathrm{HCl}=2{\mathrm{FeCl}}_{3}+{3H}_{2}O$$

(6) $${\mathrm{Al}}_{2}O_{3}+6\mathrm{HF}=2{\mathrm{AlF}}_{3}+{3H}_{2}O$$

As shown in Table 4, increasing the reaction time in the multicomponent mixed-acid system increased the Al content from 15.23 to 16.53 μg/g and the Fe content from 0.15 to 0.21 μg/g, while decreasing the purity from 99.9964% to 99.9961%. HF preferentially etched the surface structure of quartz sand, and this etching action significantly improved the permeation and diffusion of acid solutions such as HCl, HNO_3_, and HF in SiO_2_. As a strong oxidizing agent, HNO_3_ oxidized Fe^2+^ to Fe^3+^ (Equation (7)), a reaction well documented in the literature. The generated Fe^3+^ can then be complexed by Cl^−^ from HCl to form the soluble [FeCl_4_]^−^ complex ion, with a complexation constant of 1.5 (Equation (8)) [21]. However, ICP-MS results show that Fe content slightly increased (from 0.15 to 0.21 μg/g) when the leaching time was prolonged from 5 to 8 h. We infer that this unexpected rise may be due to a decrease in the solubility of Fe species over time, which could lead to secondary precipitation or adsorption of Fe-bearing colloids (e.g., Fe(OH)_3_) onto the quartz surface [22]. Regarding Al, its content also increased from 15.23 to 16.53 μg/g. Al^3+^ formed a stable complex with F^−^, such as [AlF_6_]^3−^, as shown in Equation (9), with a complexation constant of 19.7 (log β6 ≈ 19.7), which, while promoting dissolution initially, may eventually lead to the formation of sparingly soluble fluoroaluminate precipitates that hinder further leaching [23]. These mechanistic proposals are based on established coordination chemistry and are offered as plausible explanations for the observed trends.

(7) $${3\mathrm{Fe}}^{2+}+4H^{+}+{\mathrm{NO}}_{3}^{-}\to 3{\mathrm{Fe}}^{3+}+\mathrm{NO}↑+{2H}_{2}O$$

(8) $${\mathrm{Fe}}^{3+}+4{\mathrm{Cl}}^{-}\to {\left[{\mathrm{FeCl}}_{4}\right]}^{-}\;\;\;\log\beta_{4}\approx 1.5\;(25\;{}^{\circ}C)$$

(9) $${\mathrm{Al}}^{3+}+6F^{-}\to {\left[{\mathrm{AlF}}_{6}\right]}^{3-}\;\;\;\log\beta_{6}\approx 19.7\;(25\;{}^{\circ}C)$$

**Figure 7:**
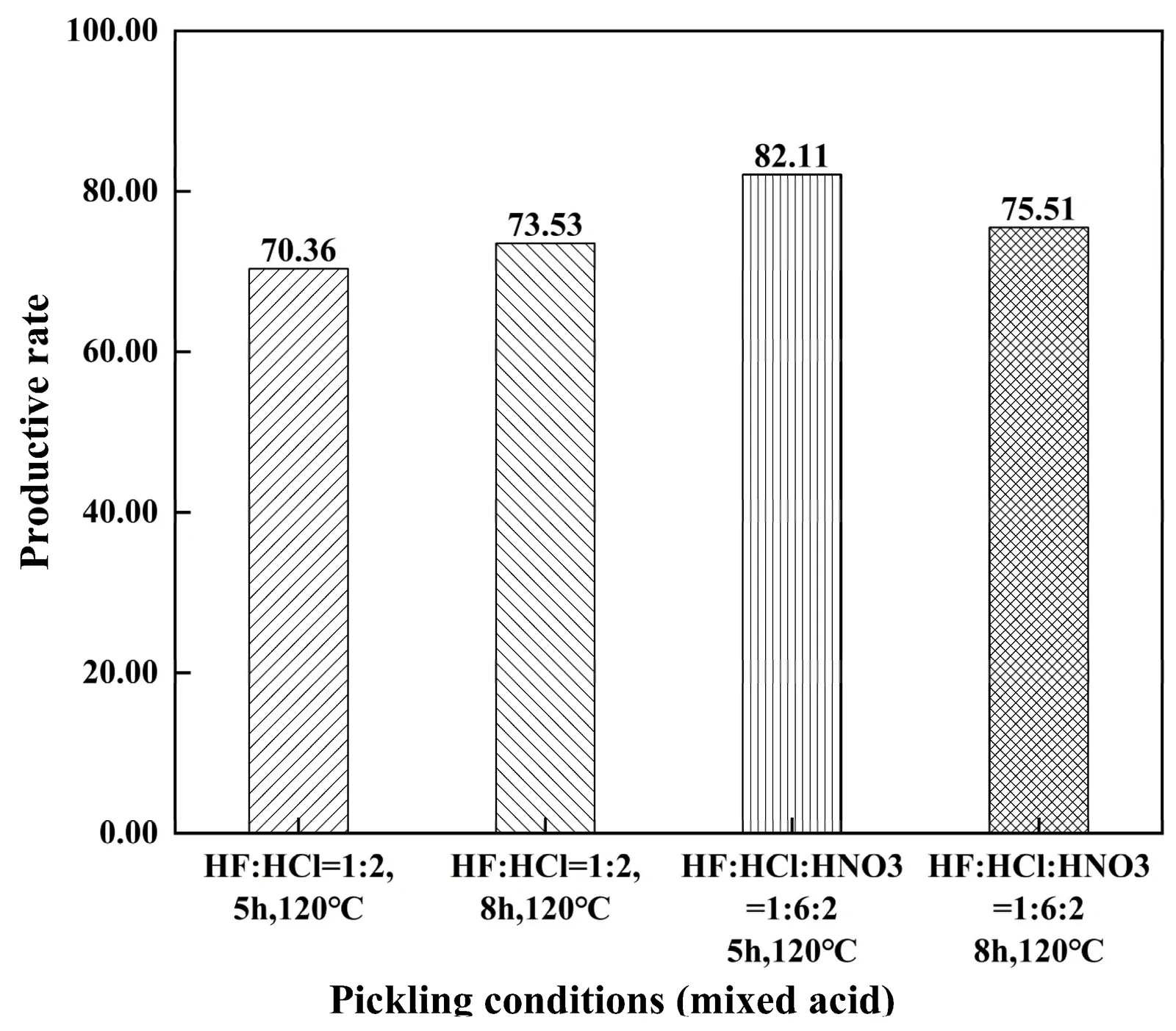
Yield of quartz sand under mixed-acid conditions.

The comparative yields of quartz sand under mixed-acid conditions are presented in Figure 7. The data showed that extending the reaction time from 5 h (yield: 70.36%) to 8 h in the HF:HCl = 1:2 system produced only a marginal increase in yield to 73.53%. This limited improvement over time suggested that the active species were almost completely consumed after 5 h or that accumulated intermediates potentially inhibited the reaction. In contrast, the HF:HCl:HNO_3_ = 1:6:2 system achieved a substantially higher yield of 82.11% after 5 h. This improvement was attributed to the strong oxidizing capacity of HNO_3_, which disrupted metal–impurity complexes within the quartz matrix. Specifically, HNO_3_ released ions such as Fe^2+^ and Al^3+^ from encapsulation in the silicate tetrahedral network through redox pathways, subsequently converting them into soluble nitrates and accelerating the dissolution kinetics of the impurities. However, the yield unexpectedly decreased to 75.51% when the reaction time was prolonged to 8 h. This decrease could hypothetically result from intensified nitrification side reactions (Equation (10)), decomposition of products under prolonged high-temperature conditions (Equation (11)), and condensation/reorganization of silanol groups during extended exposure to acidic conditions (Equation (12)) [21]. Overall, the HF:HCl:HNO_3_ ratio of 1:6:2 demonstrated optimal efficiency after 5 h. Compared with the method reported in the reference, the present approach substantially reduced the introduction of fluorides, thereby mitigating the difficulties and environmental risks associated with subsequent waste acid treatment and considerably reducing operational safety hazards [11]. Because HF and HNO_3_ are generally the most costly components of the leaching agents, substantially reducing their consumption effectively lowered the raw material costs [24].

(10) $$4{\mathrm{HNO}}_{3}\to 4{\mathrm{NO}}_{2}+{2H}_{2}O+O_{2}\;k(120\;{}^{\circ}C)\approx 0.015\;\min^{-1}$$

(11) $$H_{2}{\mathrm{SiF}}_{6}\to {\mathrm{SiF}}_{4}↑+2\mathrm{HF}↑$$

(12) $$2{\mathrm{SiO}}_{4}^{4-}+2H^{+}\to {\mathrm{Si}}_{2}O_{7}^{6-}+H_{2}O$$

To assess economic viability, the reagent costs of the optimal single-acid and mixed-acid schemes were compared using analytical-grade reagent prices. The single-acid scheme required 70 mL of HF (40% *w*/*w*) per 20 g of raw sand, corresponding to a reagent cost of approximately 5.60 CNY per batch (equivalent to 280 CNY per kg sand). In contrast, the mixed-acid scheme, with HF:HCl:HNO_3_ = 1:6:2 and a total volume of 60 mL per 20 g sand, consumed only 6.67 mL HF, 40.00 mL HCl, and 13.33 mL HNO_3_, resulting in a total reagent cost of approximately 2.06 CNY per batch (103 CNY per kg sand). This corresponded to a 63% reduction in reagent expenditure compared with the single-acid route, primarily because HF consumption was reduced by 90% and HF was the most expensive reagent. Moreover, the mixed-acid scheme provided a higher yield, further increasing the effective output per unit mass of raw sand. Collectively, these results demonstrated that the mixed-acid leaching protocol was not only technically effective but also substantially more cost-competitive for potential industrial application.

### 3.3. Mechanism Analysis

**Figure 8:**
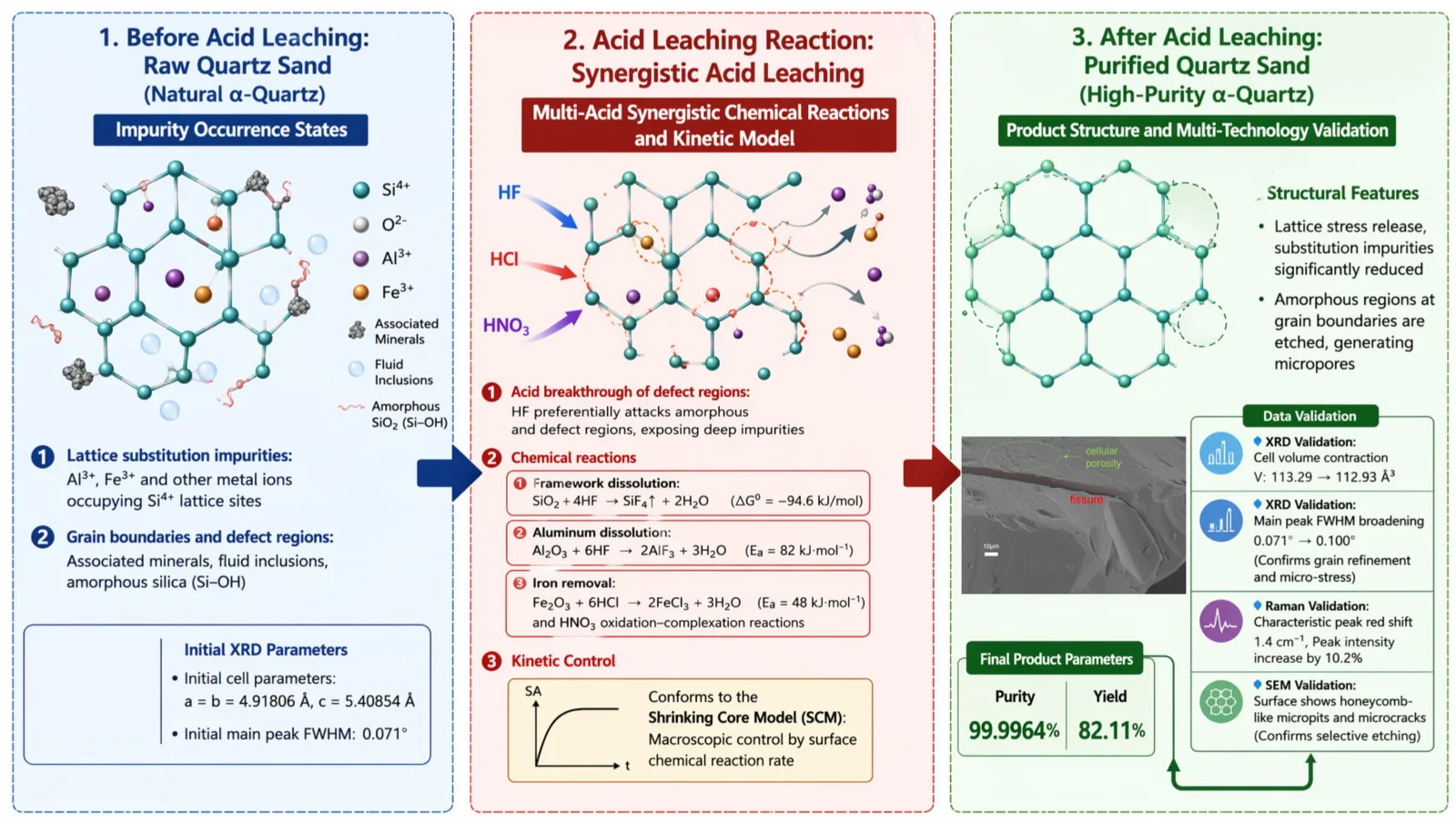
Schematic illustration of the quartz sand acid-leaching mechanism.

The schematic illustration of the synergistic purification mechanism of quartz sand during mixed-acid leaching is presented in Figure 8. The original quartz sand contains lattice-substituted impurities (such as Al^3+^ and Fe^3+^ replacing Si^4+^) and a noncrystalline Si–O network. Through specific chemical reactions, the mixed-acid system preferentially attacks lattice defects and grain boundaries following the shrinking-core model. The resulting structural changes include lattice contraction, lattice distortion, and grain refinement, changes in the bonding environment, and surface microetching, ultimately producing quartz with a purity of up to 99.9964% and a high recovery rate.

### 3.4. XRD Phase Analysis

**Figure 9:**
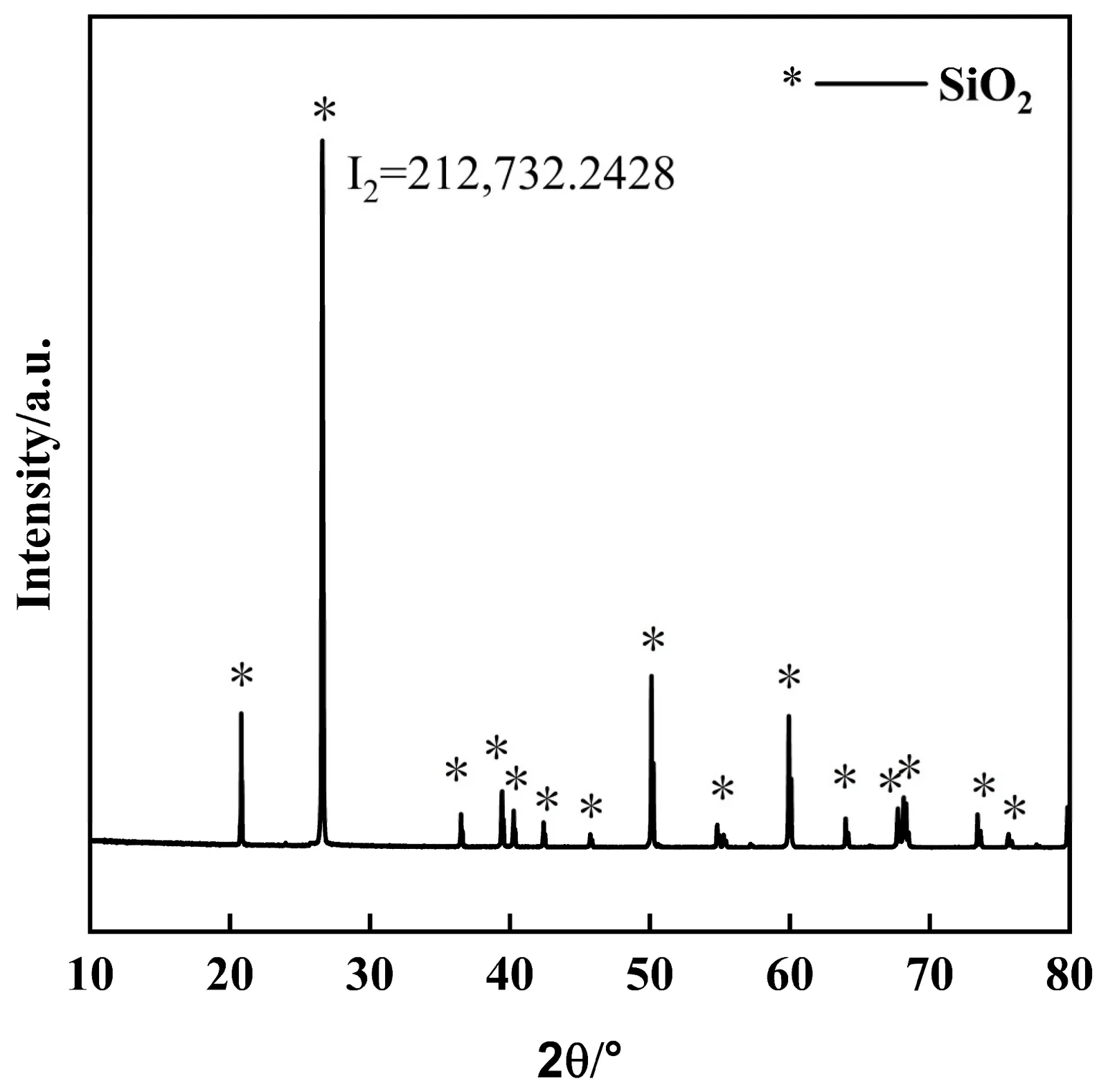
XRD pattern of quartz sand after acid leaching.

The XRD pattern of quartz sand after acid leaching under the optimized mixed-acid conditions is presented in Figure 9. To rigorously examine the structural evolution, unit-cell refinement and profile fitting were performed for the leached sample. The refinement results directly confirm that the primary α-quartz phase (space group P3_2_21, hexagonal system) remained stable, with peak positions consistent with those of the raw sand. Specifically, the refined lattice parameters of the acid-leached quartz were a = b = 4.91234 Å, c = 5.40362 Å, and V = 112.93 Å^3^, with high fitting precision (SD of Fit = 0.0296°). Notably, compared with the raw sand (V = 113.29 Å^3^), the leached sand exhibits a directly measurable lattice contraction of 0.36 Å^3^. This contraction is interpreted as evidence for the effective removal of metal impurities occupying lattice defects and the amorphous Si–O network. Consistent with this finding, the FWHM of the (101) peak at 26.6° is observed to broaden from 0.071° to 0.100° after leaching, indicating nanoscale grain refinement and dissolution of the amorphous surface layers. Furthermore, the absolute peak area of the quartz (101) plane increased more than 30-fold, while its intensity increased nearly 40-fold. Despite this increase in absolute peak intensity, the relative contribution of the characteristic quartz peak intensity to the total spectral intensity decreased from 46.52% to 31.76%. Based on these quantitative XRD observations, it is proposed that acid leaching preferentially attacked amorphous regions and crystal defects, thereby substantially improving the overall crystalline quality and purity of quartz while preserving the structural integrity of the α-quartz network [25].

### 3.5. Raman Spectroscopy Analysis

**Figure 10:**
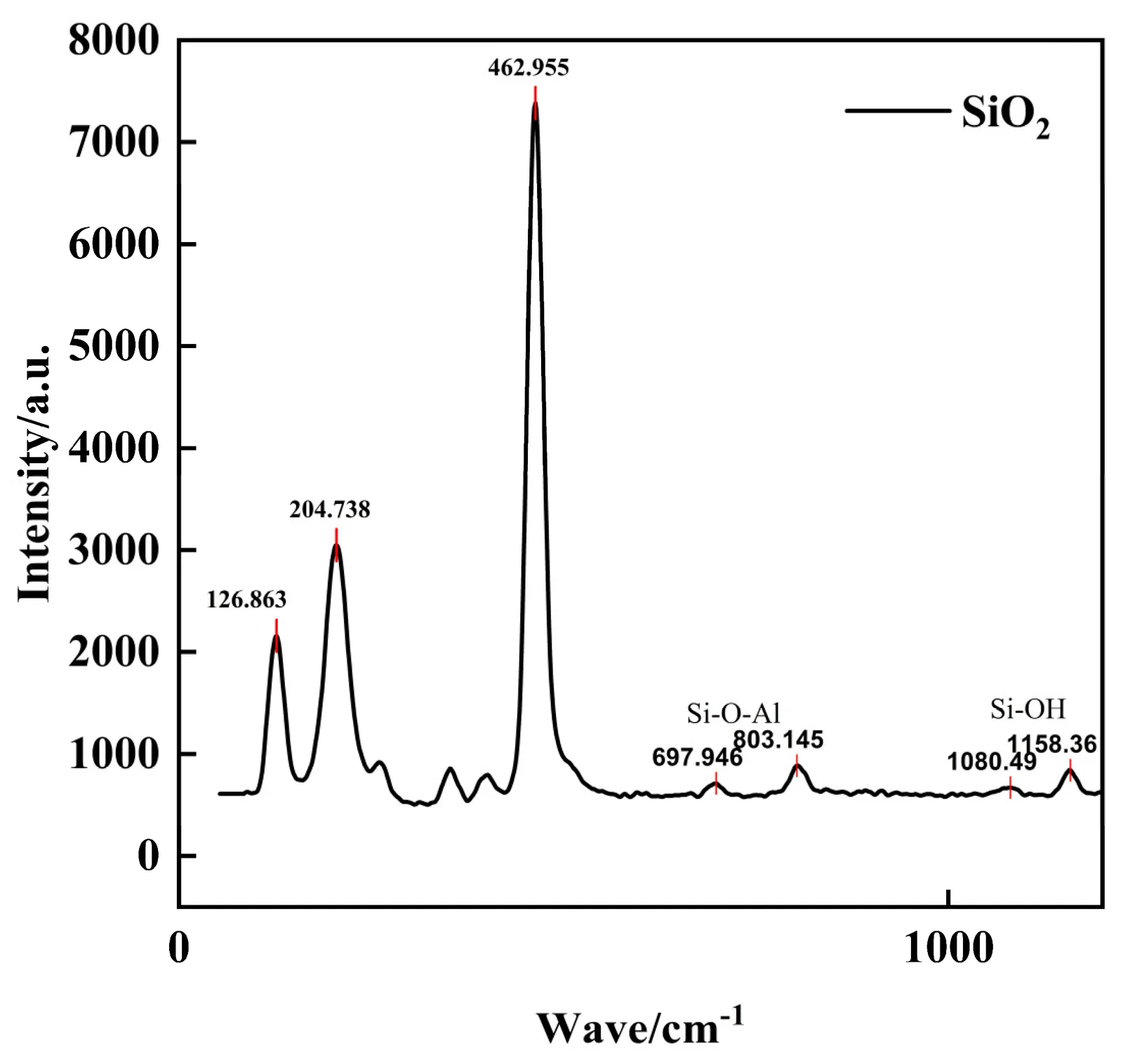
Raman spectra of quartz sand after acid leaching.

The Raman spectra of quartz sand acid-leached under the optimized mixed-acid conditions are presented in Figure 10. To establish a comprehensive structural correlation, these spectroscopic results were analyzed together with the aforementioned refined XRD parameters. Compared with raw quartz, the peaks at 126.863 and 462.955 cm^−1^ were assigned to the symmetric stretching modes of Si–O–Si, whereas the peak at 204.738 cm^−1^ corresponded to Si–O bending vibrations. Quantitative analysis reveals that the intensities of these quartz-related peaks increased by 10.2% compared with those of the raw quartz sand, indicating that the reduced impurity contents improved the purity of the SiO_2_ structure. In addition, these quartz-related peaks shifted by 1.4 cm^−1^ toward lower wavenumbers. This redshift was intrinsically associated with the 0.36 Å^3^ volume contraction observed during XRD unit-cell refinement, suggesting that removal of interstitial metal impurities released localized stress in the Si–O–Si network and consequently improved the Si–O bonding environment. Feldspar-associated Si–O–Al vibrations were detected at 697.946 and 803.145 cm^−1^, together with Si–OH bending vibrations at 1080.49 and 1158.36 cm^−1^. The persistence of these peaks after acid leaching suggests that some Al-containing impurity phases are resistant to the current leaching conditions, a finding that is consistent with the ICP-MS results showing residual Al content of 15.23 μg/g [26].

### 3.6. Microscopic Morphology Analysis

**Figure 11:**
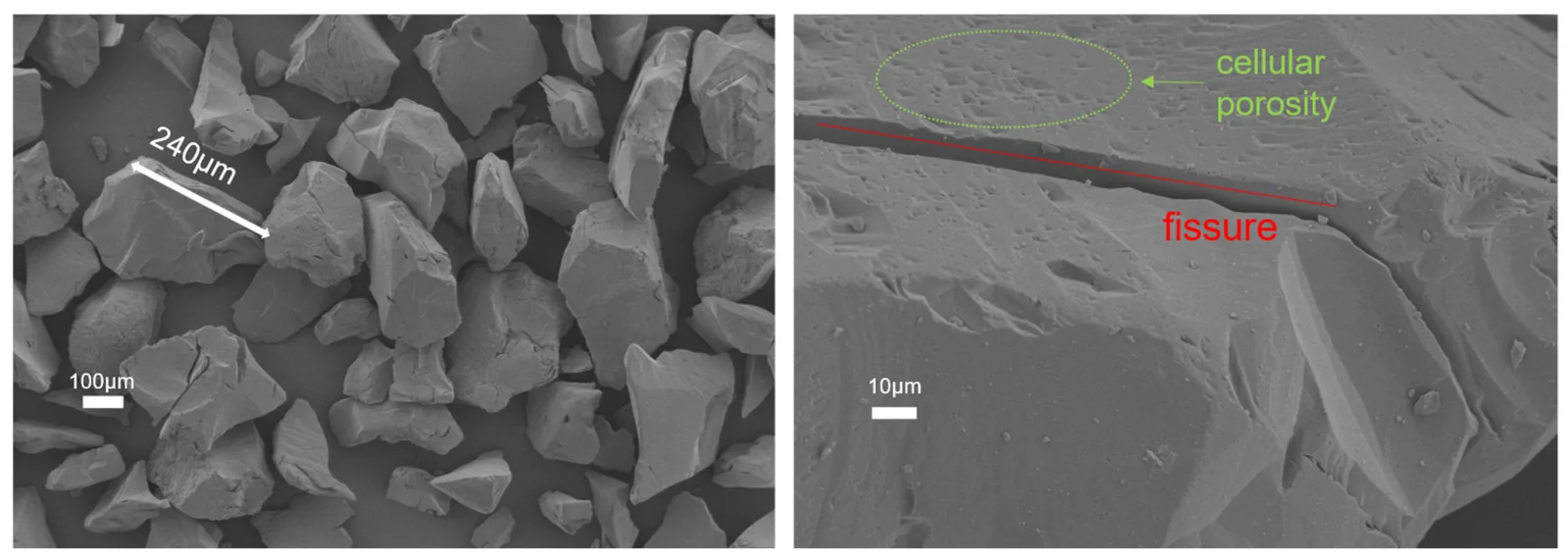
Microscopic morphology of quartz sand after acid washing.

The microscopic morphology of quartz sand after acid washing under the optimized mixed-acid conditions is shown in Figure 11. The quartz sand mainly comprised angular, block-like particles within a size range of 0.1–0.24 mm. This morphology resulted from the selective dissolution behavior of HF, which preferentially etched defective regions along the grain boundaries, causing localized dissolution and forming honeycomb-like pits [27]. Importantly, these surface morphological characteristics were consistent with the XRD profile-fitting results, in which the FWHM of the (101) peak increased from 0.071° to 0.100°. High-concentration acid solutions induced the release of internal stress within the quartz, promoting the propagation of elongated cracks along crystallographic orientations [28]. Combining the quantitative XRD and Raman results, these morphological observations confirmed that HF preferentially attacked defect sites and released impurities while inducing controlled surface etching. This synergistic purification mechanism substantially improved the surface purity and crystallinity of α-quartz while maintaining its overall structure.

## 4. Conclusions

This study proposed a mixed-acid purification route for quartz sand that simultaneously optimized purity, yield, and environmental compatibility. The main conclusions are as follows:

(1) Under single-acid leaching, the optimized conditions of 70 mL HF and a treatment duration of 2 h provided the highest purification efficiency for quartz sand. The single-acid treatment reduced the Al and Fe contents to 15.77 and 0.22 μg/g, respectively, corresponding to an impurity removal rate of 90%. The process yield reached 79.52%, producing SP1 quartz sand with a final purity of 99.9963% (4N6 level).
(2) Under mixed-acid leaching, an HF:HCl:HNO_3_ volume ratio of 1:6:2 and a reaction duration of 5 h provided the optimal purification efficiency for SP1 quartz sand. This treatment reduced the Al and Fe impurity contents to 15.23 and 0.15 μg/g, respectively, corresponding to an impurity removal rate of 90%. The process yield reached 82.11%, producing quartz sand with a final purity of 99.9964% (4N6 grade).
(3) Comprehensive analysis of the XRD patterns, Raman spectra, SEM micrographs, and ICP-MS measurements obtained before and after acid leaching confirmed the substantial effect of the purification process on quartz sand. The purity grade of the quartz sand increased from 3N6 to 4N6. However, impurity ions such as K^+^, Ca^2+^, and Al^3+^ remained detectable, indicating that further purification through flotation and roasting is required for their thorough removal. The persistence of Raman peaks at 697.946 and 803.145 cm^−1^, corresponding to Si–O–Al vibrations, indicated that some Al-containing impurities were resistant to conventional acid-leaching treatment.
(4) HF selectively etched the quartz matrix, enlarged the surface pores, and generated characteristic honeycomb-like pit structures. This morphological evolution facilitated the oxidation reactions of HNO_3_ and complexation reactions of HCl. Meanwhile, the irreversible reaction between HF and SiO_2_ inherently limited the process yield and decreased the quartz sand recovery from 79.52% to 79.05% in the single-acid leaching systems.
(5) On a laboratory scale, the mixed-acid process achieved both a higher yield (82.11%) and a substantially lower reagent cost (~63% reduction vs. single-HF treatment). However, the practical applicability of this protocol to other quartz sources will depend on their mineralogical characteristics; for ores with significant lattice-substituted impurities or different gangue assemblages, re-optimization of parameters is indispensable. Furthermore, the industrial viability of the process remains unconfirmed, as upscaling would entail substantial engineering costs, and the nitrate-laden effluent from HNO_3_ decomposition requires additional treatment steps not validated in this study. Therefore, the proposed scheme should be regarded as a preliminary concept rather than a proven industrial solution, and pilot-scale validation with diverse quartz sources, along with a full life-cycle assessment, is essential before any scale-up.

## Figures and Tables

**Table 1:** Detailed contents of impurity elements in raw SP1 quartz sand.

Element Content Details/μg/g											
Ca	Na	K	Fe	Al	Li	Mn	Cu	Ti	B	Co	Cr
25.77	60.04	39.62	5.12	122.40	0.65	0.38	0.04	1.35	0.00	0.02	0.06

**Table 2:** List of Experimental Conditions.

Acid-Leaching Conditions (Volume Concentration in %)	Dosage/Volume Ratio (mL)	Time (h)	Temperature (°C)
HF (55%)	70 mL	2/3/4	120
HF (55%)	80 mL	2/3/4	120
HF (55%) + HCl (36%~38%)	1:2 (20 mL:40 mL)	5/8	120
HF (55%):HCl (36%~38%):HNO_3_ (65%)	1:6:2 (6.67 mL:40 mL:13.33 mL)	5/8	120

**Table 3:** Detailed elemental contents under the optimal single-acid conditions.

Optimal Single-Acid Conditions: Elemental Content (μg/g)										
Ca	Na	K	Fe	Al	Li	Mn	Cu	Ti	Cr	Purity
0.50	0.64	0.65	0.22	13.77	0.50	0.03	0.03	1.16	0.01	99.9963%

**Table 4:** Optimal mixed-acid composition details.

Optimal Mixed-Acid Composition Details (μg/g)										
Ca	Na	K	Fe	Al	Li	Mn	Cu	Ti	Cr	Impurity
1.26	2.84	0.82	0.15	15.23	0.63	0.05	0.03	1.15	0.00	99.9964%

## Data Availability

The original contributions presented in this study are included in the article. Further inquiries can be directed to the corresponding authors.
